# Relationship between smoking status and muscle strength in the United States older adults

**DOI:** 10.4178/epih.e2020055

**Published:** 2020-07-28

**Authors:** R. Constance Wiener, Patricia A. Findley, Chan Shen, Nilanjana Dwibedi, Usha Sambamoorthi

**Affiliations:** 1 Department of Dental Practice and Rural Health, West Virginia University, School of Dentistry, Morgantown, WV, USA; 2 Rutgers University School of Social Work, New Brunswick, NJ, USA; 3 Department of Surgery, Department of Public Health Sciences, Penn State College of Medicine, Hershey, PA, USA; 4 Department of Pharmaceutical Systems and Policy, West Virginia University School of Pharmacy, Morgantown, WV, USA

**Keywords:** Hand grip, Smoking, Muscle strength, Older adults

## Abstract

**OBJECTIVES:**

Muscle strength in older adults is associated with greater physical ability. Identifying interventions to maintain muscle strength can therefore improve quality of life. The purpose of this study was to evaluate whether current or former smoking status is associated with a decrease in muscle strength in older adults.

**METHODS:**

Data from the Health and Retirement Study from 2012-2014 were analyzed with regard to maximum dominant hand grip strength, maximum overall hand grip strength, and smoking status (current, former, or never). Unadjusted linear regression was conducted. Other factors known to be related to strength were included in the adjusted linear regression analyses.

**RESULTS:**

For maximum grip strength, the regression coefficient was 4.91 for current smoking (standard error [SE], 0.58; p<0.001), 3.58 for former smoking (SE, 0.43; p<0.001), and 28.12 for never smoking (SE, 0.34). Fully adjusted linear regression on the relationship between dominant hand grip strength and smoking did not yield a significant result. The factors significantly associated with dominant hand grip strength were male sex, younger age, a race/ethnicity of non-Hispanic White or non-Hispanic Black, higher income, morbidity of ≤1 condition, no pain, and moderate or vigorous exercise more than once a week.

**CONCLUSIONS:**

Muscle strength in older adults was not associated with smoking status in the adjusted analysis.

## INTRODUCTION

Age-related loss of skeletal muscle mass (sarcopenia) is a public health concern [1-3]. Sarcopenia has many impacts on activities of daily living, from minor inconveniences (e.g., difficulty opening jars) to major limitations on physical health. Loss of muscle strength is a risk factor for incident cardiovascular disease [4,5] and all-cause mortality [6,7]. As the United States population ages, maintaining muscle mass and strength will be important to improve health and quality of life, increase independence, and decrease healthcare expenditures among this population.

To better understand the factors associated with sarcopenia and to determine targets for intervention, a reliable and valid muscle strength test is needed. One such test involves hand grip strength (HGS), which is correlated with other valid measures of muscle strength in adults [8] and with upper body strength in children [9]. HGS and knee extension strength have been demonstrated to have Pearson correlation coefficients of r = 0.55 to r = 0.89 and factor loadings of 0.85 to 0.91 [8]. HGS has also been shown to be a biomarker of aging [10]. A 5-kg decrease in HGS was found to be associated with a higher hazard ratio for all-cause mortality [11]. HGS cut points of 37 kg for older males and 21 kg for older females have been shown to be associated with mobility limitations [12].

In older adults, it is important to distinguish between sarcopenia (normal loss of muscle mass due to aging) and the preventable loss of muscle mass due to pathology [13], biopsychosocial factors, and/or behavior. One factor that may impact muscle strength in older adults is tobacco use, a major public health concern known to have negative health consequences. Approximately 15.5% of United States adults are current smokers. In the United States, more than 480,000 United States smoking-related deaths occur annually [14], and 87% of lung cancer deaths, 32% of coronary heart disease deaths, and 79% cases of chronic obstructive pulmonary disease can be attributed to smoking. Smoking affects overall health, the immune system, and quality of life. It is a risk factor for diabetes mellitus, rheumatoid arthritis, and poor birth outcomes [15].

Although many health consequences of tobacco use have been studied, limited research exists regarding the effect of tobacco on human muscle. Researchers using an in vitro animal model showed that solubilized tobacco smoke induced aortic smooth muscle cell death, and in vivo tobacco smoke caused elastin muscle fibers to break without an inflammatory infiltrate in mice [16]. In another in vivo mouse study, long-term smoke exposure resulted in systemic inflammation and subsequent muscle decline with a reduction in type I muscle fibers and atrophy of type II muscle fibers [17].

In a study of older smokers, relative to non-smokers, smokers demonstrated decreased cross-sectional area of type I muscle fibers, more oxidative fiber atrophy, increased glycolytic capacity, and reduced expression of nitric oxide synthase [18]. In a study of healthy, young (18- to 45-year-old) smokers versus non-smokers, smokers had higher levels of oxidative stress and skeletal muscle dysfunction in their dominant leg [19].

Smoking may affect muscle mass and strength through several mechanisms and pathways. As one example, smoking increases the level of carbon monoxide in the body. This interferes with respiratory and muscle proteins, including hemoglobin, myoglobin, and other proteins. Smoking impairs the delivery of oxygen to the mitochondria, leading to impaired generation of adenosine triphosphate and hampered contractile function [20]. Smoking has also been shown to impair muscle protein synthesis and to increase the expression of genes associated with impaired muscle maintenance [21]. On the contrary, however, researchers have shown that the nicotine in tobacco smoke may have immediate beneficial effects on motor skills.

For older adults, it is important to know whether smoking contributes to the loss of muscle mass and strength during the aging process. Therefore, the purpose of this study was to evaluate the relationship between muscle strength and smoking in older adults. The null hypothesis for this research was that among older adults, smoking has no significant effect on muscle grip strength, which is an indicator of overall muscle strength. The research hypothesis was that a significant difference exists between older smokers and non-smokers in muscle strength as indicated by grip strength. The rationale for such a difference is that smoking can damage muscle fibers and impair protein synthesis [18,21].

## MATERIALS AND METHODS

### Data source

The data used in this study were obtained from the Health and Retirement Study (HRS) from 2012-2014. The HRS is a nationally representative longitudinal panel study of an estimated 20,000 United States residents and their spouses. The survey began in 1992 and consisted of a cohort born between 1931 and 1941 (aged 51 to 61 years in 1992) and their spouses. Current members from this first cohort are now in their 70s and 80s [22]. The HRS also includes birth cohorts described as the Children of the Depression (born between 1924 and 1930), War Babies (born 1942 to 1947), Early Baby Boomers (born 1948 to 1953), Mid Baby Boomers (born 1954 to 1959), and Late Baby Boomers (born 1960 to 1965) [22]. For the present study, we used longitudinal data from 2012 to 2014.

### Study design

This study was a cohort study.

### Measures

#### Dependent variable

The dependent variables for this study were the maximum dominant HGS and maximum overall HGS. The handheld dynamometer was a pistol grip device used by participants in a standing position. The device was held with the participant’s arm at his or her side at a 90° angle to the floor. Participants were provided 1 practice attempt using the dynamometer followed by 2 recorded tests on each hand. If a participant could not stand, he or she completed the process while sitting [23].

Anthropometric physical measurements of hand strength were recorded in kilograms to the nearest 0.5 kg. As part of the HRS, participants squeezed a handheld dynamometer 2 times with each hand. The greater of the 2 results, both overall and for the dominant hand, were used in this study.

#### Key independent variable

The key independent variable was smoking status. The physical health file of the HRS included responses to questions about smoking. Participants were identified as current smokers if they answered in the affirmative to the question “Do you smoke now?” They were identified as never smokers if they responded negatively to the question “Have you ever smoked cigarettes? (By smoking, we mean more than 100 cigarettes in your lifetime. Do not include pipes or cigars).” Determining former smokers required accessing the data from 1992 to 2014. This was necessary because some participants had many quit attempts and may not have been current smokers, but had a smoking history.

#### Other variables

Socioeconomic factors (such as sex, age, race/ethnicity, education, socioeconomic/poverty status, living arrangements, and attendance of religious services) are known to support or impede engagement in preventive health behaviors [24] that could influence muscle mass and strength, such as exercise. Therefore, we included sex (male or female), age group (50-64, 65-69, 70-74, 75-79, or 80 years and above), race/ethnicity (non-Hispanic White, non-Hispanic Black, Hispanic, or other), education (less than a high school diploma, high school graduate, some college, or college degree and above), income relative to the federal poverty level (poor, low-income, middle-income, or high-income), and multiple morbidities (≤ 1 or ≥ 2). Multiple morbidity data were determined based on common chronic diseases of older adults that could impact muscle strength and mass. The diseases/conditions considered were arthritis, cancer, chronic obstructive pulmonary disease, diabetes, heart disease, hypertension, mental illness, stroke, and combinations thereof.

Other included factors were the level of pain (none, mild, moderate, or severe), depression (Center for Epidemiologic Studies Depression Scale score ≥ 3 or < 3), cognition (fair/poor, good, or very good/excellent), and exercise (none or moderate to vigorous more than once per week).

### Statistical analysis

We used the t-test to examine the pairwise comparisons and the F-test for variables with 3 or more categories. For multivariate analysis, we employed 3 ordinary least squares regression models for HGS. The models differed regarding the sets of variables for which they were adjusted. The first model was adjusted for sex, age, race and ethnicity; the second was further adjusted for education and income; and the third model included all of the above variables as well as pain, multimorbidity, depression, cognition, and physical activity.

Weights and HRS study design features were considered in the data analysis. Data were analyzed using SAS version 9.4 (SAS Institute Inc., Cary, NC, USA).

### Ethics statement

This study was reviewed by the West Virginia University Institutional Review Board (1910762141) and was acknowledged as a non-human subject (secondary data) analysis.

## RESULTS

Table 1 provides sample characteristics by smoking status. The study sample included 906 (16.9%) current smokers, 3,005 (56.3%) former smokers, and 1,560 (26.8%) individuals who had never smoked. Significant subgroup differences were present. Specifically, male, African Americans, those with income categorized as poor, those with less than a high school education, those with severe pain, those with depression, and those who did not exercise were relatively likely to be current smokers.

Table 2 presents a comparison of maximum hand grip and dominant HGS by individual characteristics. In this bivariate analysis, we found significant differences in almost all variables examined, including smoking status. Current smokers had significantly higher maximum grip strength and dominant HGS than never smokers before adjustment for other characteristics.

In Table 3, we provide the results of the ordinary least squares regression for HGS after controlling for the 3 different sets of variables. The model adjusted for only sex, age, and race/ethnicity showed that current smokers had significantly worse HGS than never smokers in terms of both maximum grip strength and dominant HGS. However, the association between smoking status and HGS was attenuated after adding education and income into the analysis in the second model. It remained insignificant in the model that included the full set of covariates.

## DISCUSSION

Current smokers had lower maximum and dominant HGS than non-smokers in our initial model, which was adjusted for age, sex, and race/ethnicity. We did not find a significant association between smoking and HGS after adjustment for sex, age, race/ethnicity, education, income, multimorbidity, depression, cognition, and physical activity.

Few studies exist with which to compare our study of male and female older adults. Our findings from the fully-adjusted model conflict with the result of a Korean study that was limited to male, in which smoking increased the odds of lower HGS (adjusted odds ratio, 4.58; 95% confidence interval [CI], 1.31 to 16.04) [25]. However, the wide CI of that finding suggests that using a larger sample size might yield different results. Similarly, the results of a study of middle-aged Japanese male (mean age, 43.3±13.9 years) also showed an association between smoking and decreased HGS [26]. Both of these studies differed from ours in that we included both older male and older female.

HGS is an important index of muscular function. Decreased muscle strength predisposes people to functional limitations and disability in their near future [27]. However, in this study, an additional impact of smoking upon muscle strength was not evident. This lack of association between smoking and HGS may be due to many factors. First, aging is the driving force of decreased HGS, and our study included community-dwelling older adults (both male and female). It is possible that older adults who were smokers or former smokers did have decreased muscle mass and strength, but that they were no longer community-dwelling (i.e., they may have been living in nursing homes or assisted living) or had died and therefore were not included in the study. It is also possible that unmeasured factors, such as history of moderate and vigorous physical activity from childhood to late adolescence [28], early-onset obesity, and inflammation [29], may mediate the relationship between smoking and HGS. As noted in the Introduction section, the nicotine in tobacco smoke may also have immediate beneficial effects on motor skills [21]. Lingering effects of nicotine or a similar mediator may therefore have impacted this relationship. Nevertheless, a strength of the present study is that it was a large, national study of older adults. The study presents a valid evaluation of participants, and the results tended toward the null.

While the driving force of decreased HGS—aging—is unavoidable, known healthful behaviors including exercise, restful sleep, healthy diet, and social interaction may help attenuate the process. As stated in the introduction, smoking has been shown to decrease HGS in Japanese male [26], and recent research has shown that cigarette smoking directly damages muscles in animal models [30]. We did not find an association of tobacco use with HGS, which is a measure of muscle health among older adults. As measured muscular health may vary depending on the measurement, it is recommended that muscular health in older adults be evaluated based on muscle mass, strength, and functional capacity [31]. Prospective cohort studies that measure various components of muscle mass and function in addition to strength are needed to confirm the association or lack thereof between smoking and muscular health among older adults.

Some limitations of our study must be considered. First, the diagnostic information used in the study was based on self-reports only. This could have introduced measurement bias from underreporting or over-reporting by some respondents [32]. Additionally, while we incorporated many factors into our models, mediators may exist that were not included, such as alcohol use. A healthy survivor effect is also possible, in which smokers were no longer alive to be included in the sample, as the data from this study are from older adults.

This study has some implications. First, again, aging is the driving force of decreased HGS. As aging cannot be avoided, it was hoped that preventive behaviors, such as tobacco cessation or never smoking, would have had a beneficial effect on muscle strength. While this was not found in the present study, known healthful behaviors such as exercise, restful sleep, healthful diets, and social interaction may help attenuate the process of muscle loss.

## Figures and Tables

**Table 1.** Sample characteristics by smoking status Health Retirement Study, 2012-2014^1^

Characteristics	Current smokers	Former smokers	Never smokers	χ^2^	p-value^2^
All	906 (16.9)	3,005 (56.3)	1,560 (26.8)		
Sex				78.662	<0.001
Female	498 (16.0)	1,530 (51.3)	1,069 (32.7)		
Male	408 (17.9)	1,475 (62.3)	491 (19.8)		
Age (yr)				652.430	<0.001
50-64	614 (27.6)	1,180 (62.2)	175 (10.2)		
65-69	108 (13.2)	408 (48.8)	315 (38.0)		
70-74	100 (9.5)	479 (47.0)	421 (43.5)		
75-79	54 (6.1)	427 (52.1)	349 (41.8)		
≥80	30 (3.1)	511 (62.2)	300 (34.7)		
Race/ethnicity				29.224	<0.001
White	512 (15.6)	2,055 (56.0)	1,214 (28.4)		
African American	266 (26.8)	499 (53.5)	179 (19.8)		
Latino	100 (17.7)	363 (60.5)	133 (21.8)		
Other race	28 (17.4)	88 (61.0)	34 (21.6)		
Education				85.459	<0.001
<High school	245 (23.5)	573 (54.0)	263 (22.5)		
High school	315 (18.0)	951 (52.7)	566 (29.3)		
Some college	247 (20.6)	739 (56.7)	322 (22.7)		
College	93 (7.8)	728 (61.7)	409 (30.6)		
Income				158.700	<0.001
Poor	226 (38.2)	291 (44.2)	113 (17.6)		
Low	224 (21.2)	562 (51.5)	322 (27.2)		
Middle	242 (14.4)	924 (55.1)	534 (30.4)		
High	214 (12.0)	1,228 (61.6)	591 (26.4)		
Pain				63.323	<0.001
None	507 (14.7)	1,929 (56.4)	1,074 (28.9)		
Mild	88 (16.6)	305 (58.4)	155 (25.0)		
Moderate	218 (19.7)	619 (57.0)	275 (23.3)		
Severe	85 (34.1)	140 (49.7)	44 (16.2)		
Cognition status				17.184	0.009
Excellent/very good	248 (17.0)	836 (59.4)	370 (23.6)		
Good	354 (15.5)	1,275 (56.5)	695 (28.0)		
Fair/poor	295 (19.0)	853 (52.8)	469 (28.2)		
Multimorbidity				13.392	0.001
≥2 conditions	465 (15.1)	1,759 (56.2)	949 (28.7)		
0 or 1 condition	441 (18.9)	1,246 (56.4)	611 (24.7)		
Depression				80.804	<0.001
CIDI ≥3	314 (28.6)	573 (49.7)	256 (21.7)		
CIDI <3	583 (14.0)	2,393 (57.9)	1,281 (28.0)		
Physical activity				46.466	<0.001
Moderate/vigorous activity	400 (13.5)	1,694 (58.5)	912 (28.0)		
No exercise	506 (21.7)	1,310 (53.1)	647 (25.2)		

Values are presented as number (%). CIDI, Composite International Diagnostic Interview.

1 Based on 5,471 living Health Retirement Study participants from 2012-2014 with no missing data on smoking, race, or hand grip strength.

2 The statistical significance of group differences in smoking status were evaluated with the Rao-Scott chi-square test.

**Table 2.** Weighted means and SE of hand grip strength by sample characteristics Health Retirement Study, 2012-2014^1^

Characteristics		Maximum grip strength	p-value^2^	Dominant hand	p-value^2^	Characteristics		Maximum grip strength	p-value^2^	Dominant hand	p-value^2^
ALL		30.96±0.20		27.84±0.19		Income					
Smoking status							Poor	28.47±0.66	<0.001	25.24±0.58	<0.001
	Current smoker	33.03±0.58	<0.001	29.57±0.57	<0.001		Low-income	27.76±0.51	<0.001	24.88±0.50	<0.001
	Former smoker	31.70±0.43	<0.001	28.56±0.41	<0.001		Middle-income	30.03±0.40	<0.001	26.97±0.40	<0.001
	Never smoker (Ref)	28.12±0.34		25.22±0.31			High-income (Ref)	33.43±0.30		30.17±0.29	
Sex		<0.001		<0.001		Pain					
	Female	24.02±0.26		21.36±0.25			None (Ref)	31.81±0.26		28.64±0.25	
	Male (Ref)	39.40±0.23		35.70±0.25			Mild	30.38±0.63	0.027	27.37±0.59	0.037
Age (yr)							Moderate	29.50±0.46	<0.001	26.24±0.44	<0.001
	50-64	34.41±0.26	<0.001	31.00±0.27	<0.001		Severe	27.38±0.84	<0.001	24.95±0.78	<0.001
	65-69	31.87±0.53	<0.001	28.61±0.53	<0.001	Cognition status					
	70-74	29.84±0.53	<0.001	26.97±0.51	<0.001		Excellent/very good (Ref)	32.36±0.36		29.18±0.33	
	75-79	26.97±0.46	<0.001	23.90±0.43	<0.001		Good	30.85±0.45	0.001	27.72±0.42	0.001
	≥80 (Ref)	22.14±0.40		19.88±0.39			Fair/poor	29.33±0.45	<0.001	26.29±0.45	<0.001
Race/ethnicity						Multimorbidity			<0.001		<0.001
	White (Ref)	31.06±0.22		28.04±0.21			≥2 conditions	28.76±0.35		25.79±0.34	
	African American	31.13±0.52	0.891	27.50±0.53	0.315		0 or 1 condition (Ref)	33.50±0.27		30.19±0.27	
	Latino	29.86±0.67	0.079	26.42±0.55	0.005	Depression			<0.001		<0.001
	Other race	30.97±0.78	0.910	27.38±0.75	0.382		CIDI ≥3	28.43±0.45		25.52±0.43	
Education							CIDI <3 (Ref)	31.49±0.23		28.32±0.21	
	<High school	29.50±0.60	<0.001	26.24±0.60	<0.001	Physical activity			<0.001		<0.001
	High school	29.83±0.56	<0.001	26.94±0.56	<0.001		Moderate/vigorous activity	32.34±0.26		29.20±0.25	
	Some college	31.41±0.48	0.007	28.19±0.50	0.012						
	College (Ref)	32.75±0.40		29.48±0.39			No exercise (Ref)	29.00±0.37		25.89±0.38	

Values are presented as mean±SE. SE, standard error; Ref, reference group; CIDI, Composite International Diagnostic Interview.

1 Based on 5,471 living Health Retirement Study participants from 2012-2014 with no missing data on smoking, race, or hand grip strength.

2 The statistical significance of group differences in hand grip strength were evaluated with the t-test via unadjusted ordinary least squares regression.

**Table 3.** Regression coefficients and SE of smoking status from ordinary least squares regressions on hand grip strength Health Retirement Study, 2012-2014^1^

Variables	Maximum grip strength			p-value^2^	Dominant hand grip strength			p-value^2^
	Beta	SE	t-value		Beta	SE	t-value	
Adjusted for sex, age, and race/ethnicity								
Intercept	17.081	0.309	55.35	<0.001	15.217	0.329	46.23	<0.001
Tobacco use								
Current smoker	-0.735	0.354	-2.08	0.043	-0.846	0.377	-2.24	0.029
Former smoker	-0.447	0.263	-1.70	0.095	-0.391	0.271	-1.44	0.154
Never smoker (Ref)								
Adjusted for sex, age, race/ethnicity, education, and income								
Intercept	18.475	0.441	41.93	<0.001	16.419	0.444	37.01	<0.001
Tobacco use								
Current smoker	-0.223	0.376	-0.59	0.556	-0.371	0.391	-0.95	0.347
Former smoker	-0.466	0.255	-1.82	0.074	-0.404	0.261	-1.55	0.128
Never smoker (Ref)								
Adjusted for sex, age, race/ethnicity, education, income, pain, multimorbidity, depression, cognition, and physical activity								
Intercept	19.892	0.482	41.25	<0.001	17.798	0.482	36.90	<0.001
Tobacco use								
Current smoker	0.185	0.367	0.50	0.616	0.015	0.399	0.04	0.970
Former smoker	-0.358	0.255	-1.40	0.166	-0.278	0.256	-1.09	0.282
Never smoker (Ref)								

SE, standard error; Ref, reference group.

1 Based on 5,471 living Health Retirement Study participants from 2012-2014 with no missing data on smoking, race, or hand grip strength.

2 The statistical significance of group differences in hand grip strength were evaluated with the t-test via multivariable ordinary least squares regression.
